# The SlERF4-9-SlCDF1/3-SlAEC2/SlPIN5 module regulates tomato root morphogenesis

**DOI:** 10.3389/fpls.2025.1546092

**Published:** 2025-03-28

**Authors:** ZhengFeng Fan, Li Zhang, SiQi Li, ShengQun Pang, YiBing Zhang, ChuanQiang Xu, YuDong Liu, MingFang Qi

**Affiliations:** ^1^ College of Agriculture, Shihezi University, Shihezi, China; ^2^ Key Laboratory of Special Sruits and Vegetables Cultivation Physiology and Germplasm Resources Utilization Xinjiang of Production and Construction Crops, Shihezi University, Shihezi, China; ^3^ College of Bioscience and Biotechnology, Shenyang Agricultural University, Shenyang, China; ^4^ Key Laboratory of Agricultural Biotechnology of Liaoning Province, Shenyang Agricultural University, Shenyang, China; ^5^ College of Horticulture, Shenyang Agricultural University, Shenyang, China

**Keywords:** auxin efflux carrier, cycling DOF factors, lateral root development, SlERF4-9, tomato

## Abstract

AP2/ERF transcription factors regulate plants’ growth, development, and stress responses. In this study, the seed germination rate and seedling growth were reduced in the tomato *slerf4-9* mutant. The fresh weight, drought weight, number of primary lateral roots (LRs), average root diameter, and number of root tips were also decreased in the mutant. The findings suggest that SlERF4-9 plays a significant role in root growth and development. The results of RNA-seq analysis of young roots indicated that the mutation of *SlERF4-9* did not affect the expression of genes related to auxin biosynthesis or signal transduction, but it did reduce the expression of the auxin efflux carrier genes *SlAEC2* and *SlPIN5*. Moreover, the mutation of *SlERF4-9* affected the distribution of auxin in the roots of DR5 × WT and DR5 × *slerf4-9* hybrid tomato seedlings. However, the promoters of *SlAEC2* and *SlPIN5* do not possess the GCC-box or DRE elements, suggesting that SlERF4-9 does not directly regulate their transcription. In addition, the expression levels of the two Cycling DOF Factors (CDFs) *SlCDF1* and *SlCDF3* decreased in the roots of the *slerf4-9* mutant. Moreover, the GCC-box was present in the promoters of *SlCDF1* and *SlCDF3*. Therefore, exploring the regulatory relationships between *SlERF4-9*, *SlCDF1*/*3*, and *SlAEC2/SlPIN5* will further our understanding of the molecular mechanisms of tomato root growth and development.

## Introduction

Roots are responsible for transporting water and nutrients during plant growth and development, and they can take in water and nutrients from the soil to meet the demands of the plant and enhance its tolerance to abiotic stresses. In many plants, the number of lateral roots (LRs) often determines the efficiency of water and nutrient utilization (Casimiro et al., 2003). Therefore, cultivating new varieties with enhanced and better-developed root systems has become an important goal in crop breeding.

The growth and development of plant roots cannot be separated from auxin regulation. Auxin is an important phytohormone that is involved in almost all stages of plant growth and development (Overvoorde et al., 2010; Yu et al., 2022). This phytohormone is synthesized in source tissues such as the stem tip, young leaves, cotyledons, and root tips (Ljung et al., 2001; Peret et al., 2009), and it is transported to other tissues through polar or phloem transport (Benková et al., 2003). Phloem transport relies on the concentration gradient of organic matter, while polar transport is a short-distance and unidirectional transport mode between the parenchymal cells of the coleoptile, young stem, and young roots (Blakeslee et al., 2005). During polar auxin transport, auxin efflux (PIN), and influx (AUX/LAX) carrier proteins are involved in the regulation of plant growth and development (Bennett et al., 1996; Petrasek et al., 2006; Swarup et al., 2001). In roots, AUX/LAX and PIN synergistically regulate cell differentiation by controlling the cellular level of free auxin. In *Arabidopsis*, AtAUX1 regulates cell differentiation in the lateral root cap and epidermis (De Smet et al., 2007), whereas AtLAX3 regulates lateral root emergence (Swarup et al., 2008). The polar transport of auxin has been found to be significantly reduced in the *atpin1* mutant (Okada et al., 1991). Moreover, PIN1 can transport auxin via PIN1–PIN1 dimers (Teale et al., 2021).

Ethylene, as a plant-specific hormone, is involved in fruit ripening and softening, organ senescence, flower organ development, root development, and the response to abiotic stresses (Jiroutova et al., 2018; Pieterse et al., 2009). During root development, treatment with the ethylene inhibitor 1-MCP was found to improve root elongation and increase the number of LRs in tomatoes while simultaneously repressing root penetration into the soil (Santisree et al., 2011). In the *Arabidopsis aco1* mutant, ethylene production in the root tips is reduced, and the number of LRs is increased (Park et al., 2018). These results suggest that ethylene positively regulates root gravitropism while inhibiting root elongation and lateral root development. The *AP2/ERF* regulatory genes encode specific ethylene-responsive transcription in plants, playing important roles in plant growth, development, and stress response (Jia et al., 2021; Xu et al., 2024; Yuste-Lisbona et al., 2020). In *Arabidopsis*, *AtERF070* negatively regulates the number and length of LRs (Ramaiah et al., 2014). However, in *Populus*, *PtaERF003* had been found to promote lateral root formation (Trupiano et al., 2013). Tomato *SlERF4-9* (*SlERF.H12*/*SlDREB3*) overexpression improves the root growth of tobacco and cold tolerance in tomatoes (Upadhyay et al., 2017; Wang et al., 2019; Zhang et al., 2022). However, the mechanism by which *SlERF4-9* regulates root development remains unclear.

In addition to the AP2/ERF family, the DNA binding with One Finger (DOF) family is a unique transcription factor in plants, and its members are involved in various aspects of plant growth and development, including seed germination, the photoperiodic control of flowering, vascular development, and shoot branching (Fornara et al., 2009; Gabriele et al., 2010; Konishi and Yanagisawa, 2007; Zou et al., 2013). In bananas, MaDof23 regulates fruit ripening by interacting with MaERF9 (Feng et al., 2016). In *Arabidopsis*, the *AtDof5.1* mutation reduces the expression levels of the auxin-responsive genes *IAA6* and *IAA19* and alters auxin homeostasis in the root tips (Kim et al., 2010). Therefore, there may be a relationship between AP2/ERF, DOF, and auxin during plant root development.

In this study, we found that root growth was significantly reduced in the *slerf4-9* mutants. RNA-seq analysis of young roots revealed that the expression levels of genes involved in auxin biosynthesis and signal transduction were not significantly altered. However, the expression of the auxin efflux carriers *SlAEC2* and *SlPIN5* was significantly decreased in *slerf4-9* roots, suggesting that SlERF4-9 may regulate root growth and development by modulating their transcription.

Interestingly, while the *SlAEC2* and *SlPIN5* promoters lack the GCC-box or DRE elements, they contain multiple DOF protein binding elements, indicating that their reduced expression may be an indirect consequence of the SlERF4-9 function. Furthermore, we observed the significant downregulation of Cycling DOF Factors (*SlCDF1* and *SlCDF3*) in the roots of *slerf4-9*, whose promoters bear GCC-box elements, suggesting a possible regulatory link between SlERF4-9 and SlCDF1/3 in the control of *SlAEC2* and *SlPIN5*.

These findings suggest a regulatory module, where SlERF4-9 may influence root development through the modulation of SlCDF1/3, which in turn could affect auxin transport via *SlAEC2* and *SlPIN5* gene regulation. Further investigations into these regulatory interactions will shed light on the role of SlERF4-9 on auxin transport during root growth.

## Materials and methods

### Plant materials

All tomato plants were derived from the *Ailsa Craig* (AC) strain. The *slerf4-9* knockout mutants were created using CRISPR/CAS9 technology with AC as the background material. The AC wild-type (WT) and *slerf4-9* mutant lines #2 and #31 were used in this study. The tomato plants were grown for 15 and 30 days under 25°C 16 h/15°C 8 h day/night conditions. The roots of the 30-day seedlings were scanned and analyzed using WinRHIZO Pro root analysis software (Epson Perfection V850 Pro), and the soil volume analysis parameters were set to 1.0 cm^3^. To investigate auxin transport in tomato roots, the pollen of tomato DR5 was used to pollinate the stigma of the WT and *slerf4-9* mutant to generate DR5 × WT and DR5 × *slerf4-9* hybrid seeds. These seeds were then used in a GUS (β-glucuronidase) staining assay.

### Seed germination assay

Thirty seeds of the WT and *slerf4-9* mutant lines #2 and #31 were heated at 55°C for 15 min, placed in a culture dish with moist filter paper, and germinated at 25°C for 7 days. In addition, 100 mg of the young roots of seeds germinated for 7 days was gathered for transcriptomics analysis. Three biological replicates were set for each treatment.

### DNA and RNA extraction

A total of 100 mg of the fresh leaves of the WT tomatoes was used to extract DNA using a Plant Genomic DNA Kit (DP305-03; Tiangen, Beijing, China) to subsequently amplify the promoters of *SlCDF1* (Solyc03g115940), *SlCDF3* (Solyc06g069760), *SlAEC2* (Solyc02g082450), and *SlPIN5* (Solyc01g068410). The young roots of the WT and *slerf4-9* mutants were used to extract total RNA using TRIzol Reagent (Invitrogen, Carlsbad, CA, USA). The RNA was used for transcriptomics sequencing, quantitative real-time PCR (qRT-PCR), and the amplification of the Coding sequence (CDS) fragments of *SlERF4-9*, *SlCDF1*, and *SlCDF3*.

### Subcellular localization assay

To determine whether SlERF4-9 had potential nuclear regulatory functions, the CDS of SlERF4-9 was amplified using the primers ERF4-9 F/R (the primers are listed in **Supplementary Table S1**) and inserted between the cleavage sites *Bam*HI and *Sal*I in the pCAMBIA1300 vector using an In-Fusion PCR Cloning Kit to form a *35S::SlERF4-9-GFP* recombinant vector. The recombinant vector was then transformed into *Agrobacterium* strain GV3101. Subsequently, 15 mL of a suspension of *Agrobacterium* cells with the *35S::SlERF4-9-GFP* vector (OD 1.0) was centrifuged at 4°C and 5,000 × *g* for 5 min; after this, the supernatant was removed, and the cells were resuspended in 5 mL of solution (10 mM·mL^−1^ MES, 10 mM·mL^−1^ MgCl_2_, and 20 μM·mL^−1^ AS, pH 5.6) and placed in the dark at 25°C for 3 h. Finally, the above-mentioned solution was injected into tobacco leaves, and the tobacco plants were placed in the dark at 25°C for 2 days. *Agrobacterium* cells carrying the pCAMBIA1300 vector were used as a control in this assay.

### Transcriptomics analysis

The young roots (200 mg) of the WT and *slerf4-9* mutant lines #2 and #31 from seeds germinated for 7 days were collected for transcriptomics analysis. The WT roots (young roots of AC, named WT) were analyzed as a control group, and the E2 roots (young roots of the *slerf4-9* mutant line #2, named E2) and E31 roots (young roots of the *slerf4-9* mutant line #31, named E31) comprised the experimental groups. The experiment adopted a paired design, in which the young root samples of WT, E2, and E31 were synchronously processed under the same culture conditions, with three independent biological replicates set for each genotype. When conducting paired analysis, the WT was matched one-to-one with the three biological replicates of each mutant to eliminate batch effects. Total RNA was extracted using TRIzol Reagent (Invitrogen) and employed to construct RNA-seq libraries. The sequences were read using the Illumina HiSeq 2000 platform. Agilent 2100 Bioanalyzer was used for quality control of libraries. The raw data were evaluated for quality using FastQC (v0.11.9), and low-quality bases were removed using Trimmomatic. Clean reads were aligned to the tomato reference genome (https://solgenomics.net/ftp/tomato_genome/annotation/ITAG4.0_release/) using HISAT2 (v2.2.1) and filter reads with unique alignment rate ≥90% using SAMtools (v1.12). The fragments per kilobase of exon model per million mapped fragments (FPKM) values of each gene were computed using the HiSeq (ver. 0.6.1) and DESeq Bioconductor packages. Differentially expressed genes (DEGs) were analyzed by DESeq2 (v1.34.0), and the screening criteria were |log2FoldChange| > 1 and false discovery rate (FDR) corrected p-value < 0.05. FDR calculation adopted the Benjamini–Hochberg method. Gene Ontology (GO) TermFinder (v0.4) was used to analyze the GO enrichment by a hypergeometric test based on the ITAG4.0 annotated tomato GO database, with filtering criteria of corrected p-value < 0.05 and exclusion of entries containing “unknown function”. Finally, Kyoto Encyclopedia of Genes and Genomes (KEGG) pathway analysis was performed using KOBAS (v3.0), with a pathway significance threshold of FDR < 0.05.

### Quantitative real-time PCR

Total RNA was extracted using TRIzol Reagent (Invitrogen), and cDNA was synthesized using a PrimeScript™ 1st Strand cDNA Synthesis Kit (TaKaRa, Kyoto, Japan). qRT-PCR was conducted using TB Green^®^ Premix Ex Taq™ II FAST qPCR (TaKaRa, Kyoto, Japan). The reaction conditions were as follows: 95°C for 30 s, 40 cycles of 95°C for 5 s, and 60°C for 10 s. The primers used in this assay are listed in **Supplementary Table S1**. A dissociation curve was used to validate the specificity of the primer pair. Three biological replicates were set up for this assay. The cycle threshold value of each gene was used to calculate the relative expression level of each gene using the 2^−ΔΔCT^ method (Nolan et al., 2006). In addition, the differences in expression levels were analyzed using t-tests.

### Prokaryotic expression assay

To obtain the SlERF4-9 protein, the CDS of *SlERF4-9* was amplified using the primers 6P-ERF4-9 F/R (the primers are listed in **Supplementary Table S1**) and inserted between the cleavage sites *Bam*HI and *Xho*I of the pGEX6P-1 (GST-tag) vector using an In-Fusion PCR Cloning Kit to form a *35S::SlERF4-9-GST* recombinant vector. The recombinant vector was then transformed into *Escherichia coli* competent BL21 cells (DE3). The *E. coli* cells with the *35S::SlERF4-9-GST* vector were cultivated in 50 mL of lysogeny broth liquid medium with 50 μg·mL^−1^ ampicillin at 37°C for 200 × *g* for 12 h, then supplied with 50 μL of 1 M isopropyl-beta-d-thiogalactopyranoside, and finally cultivated at 37°C for 200 × *g* for 12 h. Next, the above cells were centrifuged at 5,000 × *g* for 10 min at 4°C, and the supernatant was removed. Then, 10 mL of phosphate buffered saline (PBS) with lysozyme (1 mg·mL^−1^) was used to break down the cell walls using an ultrasonic crusher in an ice bath for 30 min. The bacterial fluid was centrifuged at 10,000 × *g* for 10 min at 4°C to separate the supernatant and precipitate. The supernatant was used to purify the SlERF4-9-GST fusion proteins using a GST-tag Protein Purification Kit (Beyotime, Shanghai, China). Finally, 20 μL of the purified protein was detected using Sodium Dodecyl Sulfate Polyacrylamide Gel Electrophoresis (SDS-PAGE).

### Electrophoretic mobility shift assay

The purified SlERF4-9-GST fusion protein was used to detect the binding ability of SlERF4-9 with the GCC-box of the SlCDF1 and SlCDF3 promoters. The biotin probes (named prob-Bio), cold probes (named prob-Cold), and mutated probes (named Mu-prob-Bio) of the P1 and P2 sites were synthesized (the probe sequences are listed in **Supplementary Table S1**). The specific binding of the protein with DNA was validated by competition with the prob-Cold (100×) and Mu-prob-Bio probes. The electrophoretic mobility shift assay (EMSA) was carried out using a Chemiluminescent EMSA Kit (Beyotime, Shanghai, China).

### Dual-luciferase transient expression assay

The promoters of *SlCDF1* (−1 to −1,500 bp upstream of ATG), *SlCDF3* (−1 to −1,487 bp upstream of ATG), S*lAEC2* (+33 to −1,502 bp upstream of ATG), and *SlPIN5* (+15 to −1,489 bp upstream of ATG) were amplified and inserted into a pGreenII-0800-Luc vector using an In-Fusion PCR Cloning Kit to form the *proSlCDF1-Luc*, *proSlCDF3-Luc*, *proSlAEC2-Luc*, and *proSlPIN5-Luc* recombinant vectors. The CDS fragments of *SlERF4-9*, *SlCDF1*, and *SlCDF3* were cloned and inserted into the pGreenII-62-SK vector to construct the *SK-SlERF4-9*, *SK-SlCDF1*, and *SK-SlCDF3* recombinant vectors, respectively. The primers used for amplification are listed in **Supplementary Table S1**. The above-mentioned recombinant vectors, alongside pGreenII-0800-Luc and pGreenII-62-SK empty vectors, were then transformed into *Agrobacterium* GV3101 competent cells. The cells (OD_600_ value = 0.8) with *SK-SlERF4-9* were combined with those of *proSlCDF1-Luc* and *proSlCDF3-Luc*; the cells with *proSlAEC2-Luc* and *proSlPIN5-Luc* were combined with those of *SK-SlCDF1* and *SK-SlCDF3*; and the cells of pGreenII-62-SK and pGreenII-0800-Luc replaced those of the corresponding recombinant vectors as negative controls. The mixtures of cells were placed in the dark at 28°C for 3 h, injected into tobacco leaves, and cultured in the dark at 25°C for 24 h. d-Luciferin potassium salt was then sprayed on the injected tobacco leaves. The fluorescent signal of the injected tobacco leaves was detected using a plant multispectral fluorescence imaging system. The luciferase was extracted using a dual-luciferase reporter assay system (Promega, Madison, WI, USA; E1910), and the activity analysis of LUC and REN was conducted utilizing a SpectraMax iD3 instrument (Molecular Devices, San Jose, CA, USA).

### GUS staining

The germinated 7-day seedlings of the DR5 × WT and DR5 × *slerf4-9* hybrid seeds in sterile water were placed in a 15-mL centrifuge tube containing 10 mL of GUS staining solution (500 mg·L^−1^ X-Gluc, 5-bromo-4-chloro-3-indolyl glucuronide) and stained at 37°C for 12 h. The staining solution was removed, and the seedlings were destained at 37°C using 70% ethanol until the green color of the cotyledons and hypocotyl had faded.

### Bioinformatics and statistical analyses

Transcriptomics data were analyzed using R (http://www.r-project.org/). All heatmaps in this study were produced using the Pvclust package (Suzuki and Shimodaira, 2006) and the pcaMethods Bioconductor package (Stacklies et al., 2007). The phylogenetic tree of the CDF proteins of tomato and *Arabidopsis* was constructed using MEGA v7.0 and CLUSTAL W. The parameters for the ML method were a + JTT matrix model with a site coverage cutoff of 50%, Poisson correction, and self-expansion value of 1,000. Conserved motifs of the CDF proteins in tomato and *Arabidopsis* were analyzed using the MEME (Multiple Em for Motif Elicitation) online software v.5.4 (https://meme-suite.org/meme/tools/meme). All data were analyzed as the mean ± standard error of three biological repeats. Statistically significant differences (p < 0.05) for the mean of each sample (Student’s t-test; *p < 0.05, **p < 0.01, and ***p < 0.001) were detected using a *post-hoc* test following an analysis of variance (ANOVA).

## Results

### *SlERF4-9* mutation reduces the germination ability of tomato seeds

**Figure 1:**
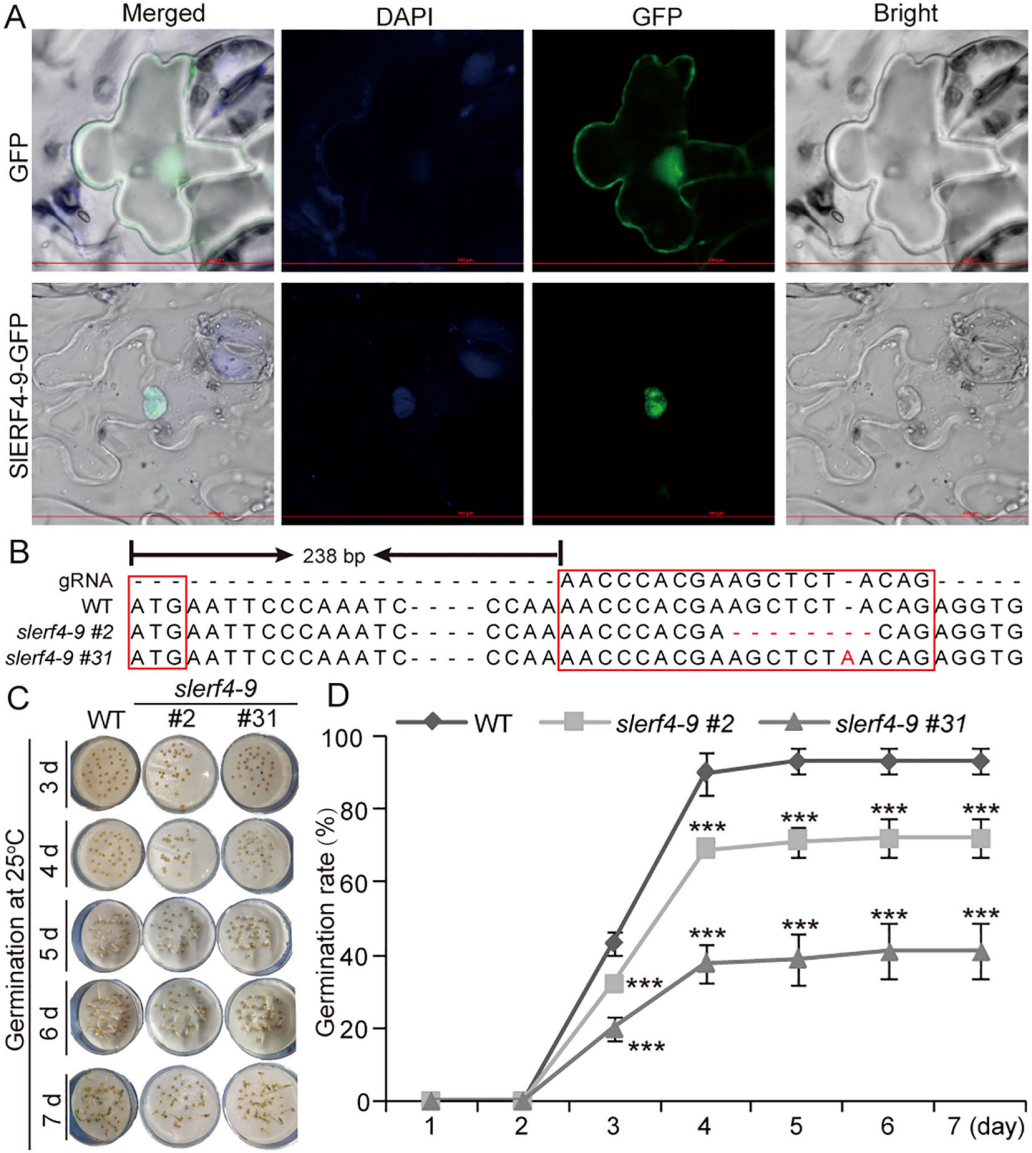
Seed germination analysis of the *slerf4-9* mutants. **(A)** Subcellular localization of SlERF4-9. **(B)** Mutation site analysis of *SlERF4-9*. **(C, D)** Germination rate analysis of *slerf4-9#2* and *slerf4-9#31* mutants. WT, tomato *Ailsa Craig* (AC). Asterisks denote significance relative to WT (Student’s t-test; ***p < 0.001).

The subcellular localization results indicated that the SlERF4-9 protein was only detected in the nucleus (**Figure 1A**). Therefore, SlERF4-9, as a nuclear transcription factor, may have a regulatory role in tomato growth and development. To explore the function of SlERF4-9, an *slerf4-9* mutant was created using CRISPR/Cas9 technology. In the *slerf4-9* mutants, the gRNA site of *slerf4-9#2* had “AGCTCTA” sequences deleted, while that of *slerf4-9#31* had an “A” inserted (**Figure 1B**). The results suggested that both *slerf4-9#2* and *slerf4-9#31* would be unable to be correctly translated to the SlERF4-9 protein. After seed germination at 25°C for 3 days, 43.33%, 32.22%, and 20% of the seeds germinated in the WT, *slerf4-9#2*, and *slerf4-9#31*, respectively. After germination for 4 days, 90%, 68.89%, and 37.78% of the seeds germinated in the WT, *slerf4-9#2*, and *slerf4-9#31*, respectively. After germination for 7 days, the germination rates of the WT, *slerf4-9#2*, and *slerf4-9#31* seeds were 93.3%, 72.2%, and 41.1%, respectively (**Figures 1C, D**). The results suggest that the *SlERF4-9* mutation reduces the germination ability of tomato seeds at 25°C.

### *SlERF4-9* mutation reduces lateral root development in tomato seedlings

**Figure 2:**
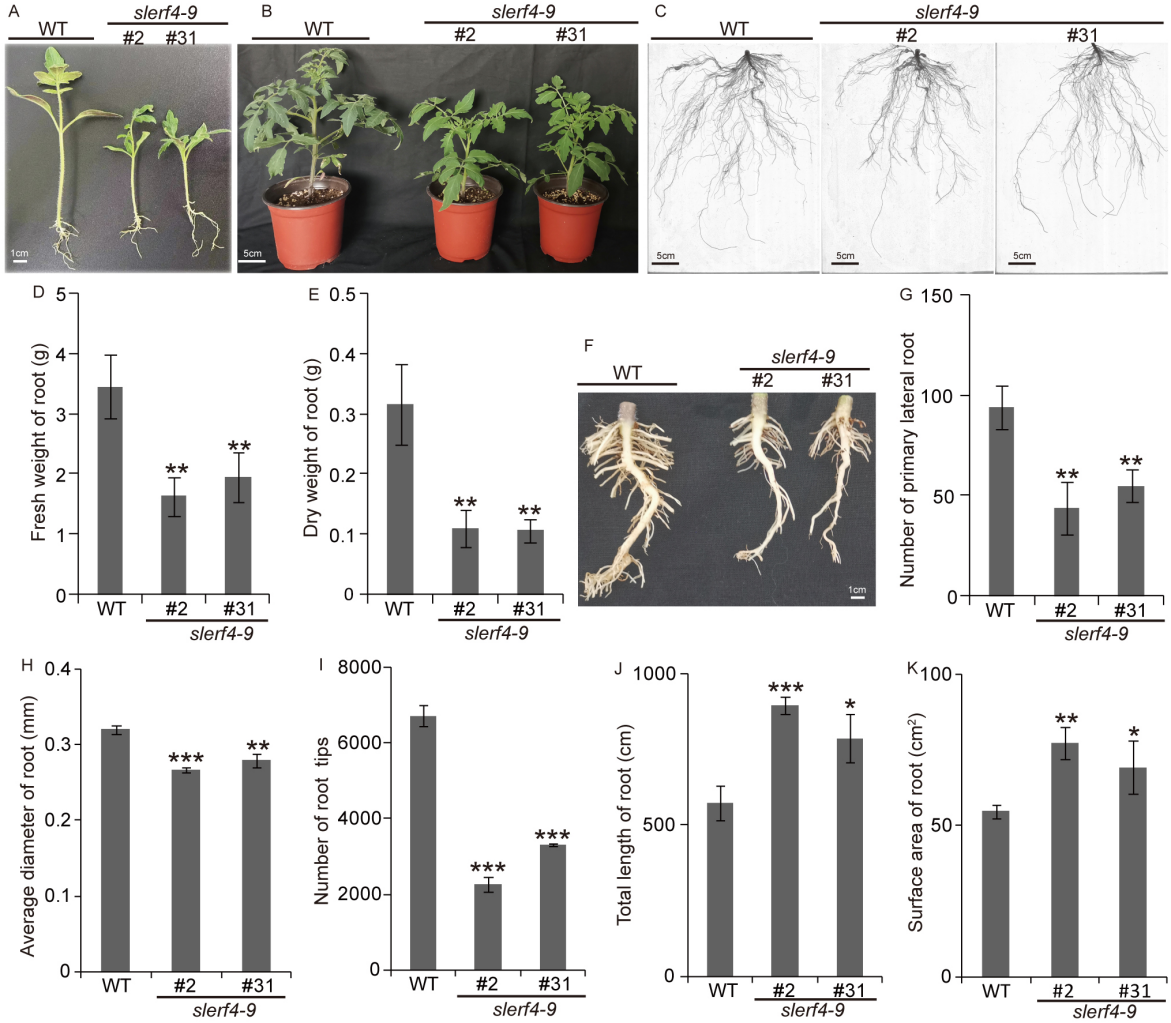
Phenotype analysis of *slerf4-9* mutants. **(A, B)** Phenotype of the growth 15- and 30-day mutant seedlings. **(C)** Complete roots of the 30-day mutant seedlings using WinRHIZo Pro root analysis software. **(D, E)** Comparison of fresh weight and drought weight of the 30-day mutant roots. **(F, G)** Comparison of primary LR number of the 30-day mutant roots. **(H)** The average diameter. **(I)** Tip number. **(J)** Total length. **(K)** Surface area of root. WT, tomato *Ailsa Craig* (AC). Asterisks denote significance relative to WT (Student’s t-test; *p < 0.05, **p < 0.01, ***p < 0.001). LR, lateral root.

After seed germination at 25°C for 16 h/16°C for 8 h (day/night) for 15 days, the WT seedlings grew faster than the *slerf4-9#2* and *slerf4-9#31* mutants (**Figure 2A**). In addition, the WT 30-day seedlings showed numerous and stronger lateral roots than the *slerf4-9* mutants (**Figures 2B, C**). The fresh weight, dry weight, primary lateral root number, average diameter, and the number of tips of the roots in the mutants were reduced compared with those of the WT (**Figures 2D–I**). However, the total length and surface area of the roots showed an opposite trend (**Figures 2J, K**). The reason for this may be that the LRs of the *slerf4-9* mutants were slender and elongated, leading to an increase in the root surface area. The results indicate that SlERF4-9 regulates tomato lateral root development.

### *SlERF4-9* affects the expression of genes related to antioxidant and plant hormone signal transduction

**Figure 3:**
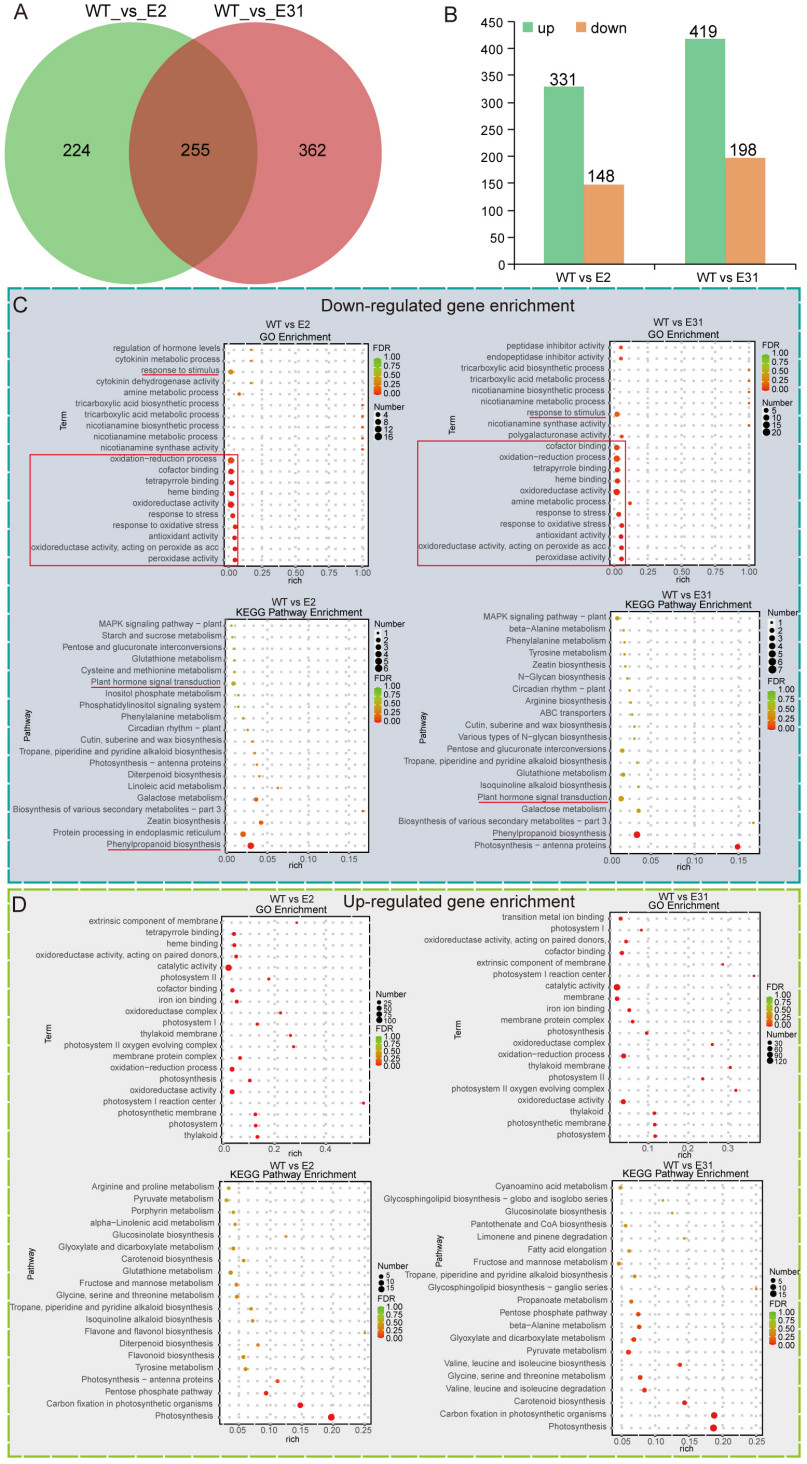
The Venn diagram, GO, and KEGG enrichment analysis. **(A)** Comparison of DEGs between WT_vs_E2 and WT_vs_E31. **(B)** Statistics on the number of upregulated and downregulated DEGs. **(C, B)** The GO and KEGG enrichment analyses of upregulated and downregulated DEGs, respectively. WT, E2, and E31, respectively indicate the 7-day seedling roots of AC, *slerf4-9#2*, and *slerf4-9#31*. GO, Gene Ontology; DEGs, differentially expressed genes.

To understand the regulatory network of SlERF4-9 in tomato root development, the young roots of WT, *slerf4-9#2*, and *slerf4-9#31* germinated for 7 days were collected and subjected to RNA-seq analysis. The transcriptome sequencing raw data were uploaded to the National Center for Biotechnology Information (NCBI) Sequence Read Archive (SRA) database (PRJNA1091043). The FPKM values of 34,075 genes in the transcriptome data were analyzed (**Supplementary Table S2**). The DEG analysis identified 479 and 617 DEGs in the WT_vs_E2 and WT_vs_E31 comparisons, respectively. There were 255 DEGs common to the WT_vs_E2 and WT_vs_E31 comparisons (**Figure 3A**). In addition, there were 331 upregulated and 148 downregulated DEGs in the WT_vs_E2 comparison and 419 upregulated and 198 downregulated DEGs in the WT_vs_E31 comparison (**Figure 3B**; **Supplementary Tables S3**-**S6**). Several downregulated DEGs were enriched in the response to stimulus and antioxidant systems in the GO analysis; plant hormone signal transduction and phenylpropanoid biosynthesis were identified by the KEGG pathway enrichment analysis of WT_vs_E2 and WT_vs_E31 (**Figure 3C**). The results suggest that the *SlERF4-9* mutation causes a decrease in the expression of genes related to hormone signal transduction and antioxidant pathways. In addition, some upregulated DEGs were enriched in catalytic activity, oxidation–reduction process, and oxidoreductase activity in the GO analysis, and in substance biosynthesis and metabolism in the KEGG pathway enrichment analysis, of WT_vs_E2 and WT_vs_E31 (**Figure 3D**). The results indicate that SlERF4-9 may negatively regulate the expression of genes related to biosynthesis and metabolism during tomato root development.

### *SlERF4-9* mutation does not affect the expression of genes related to auxin biosynthesis or signal transduction

**Figure 4:**
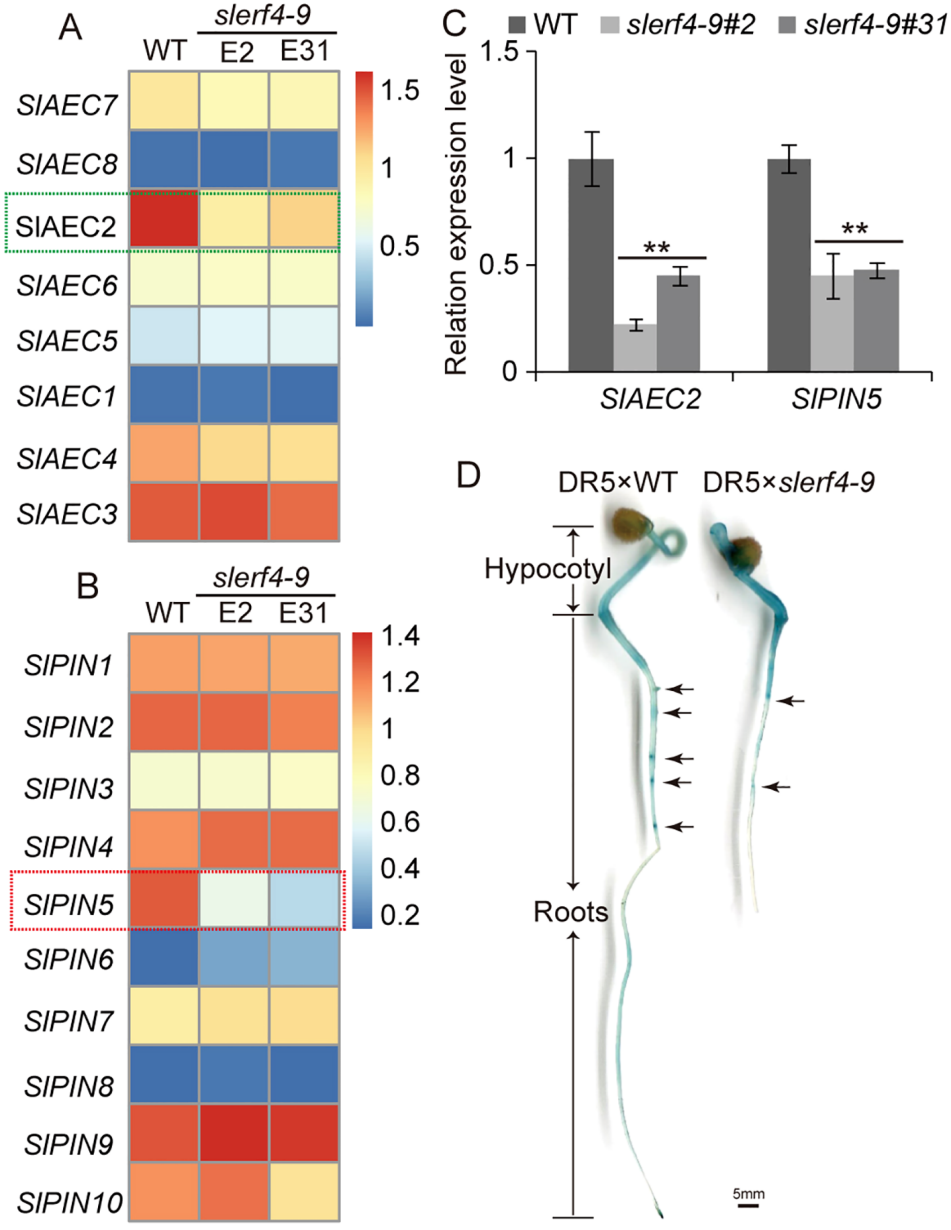
Auxin transport analysis. The FPKM value comparison of **(A)** *SlAEC* and **(B)** *SlPIN* family members. **(C)** Expression analysis of *SlAEC2* and *SlPIN5*. **(D)** GUS staining analysis of the DR5 × WT and DR5 × *slerf4-9* hybrid seed germinated seedlings. Asterisks denote significance relative to WT (Student’s t-test; **p < 0.01).

The transcriptome data demonstrated that the FPKM values of 10 *SlYUCCA* and three *SlTAA* genes in two *slerf4-9* mutants did not differ from those in the WT (**Supplementary Figures S1A, B**). This indicated that SlERF4-9 was not involved in auxin biosynthesis. In addition, the FPKM values of 22 *SlARF* and 25 *SlIAA* genes in two *slerf4-9* mutants did not differ from those of the WT (**Supplementary Figures S1C, D**). However, we found that among eight auxin efflux carrier (AEC) family members, only the FPKM value of *SlAEC2* (Solyc02g082450) significantly decreased in two *slerf4-9* mutants, while those of *SlAEC8* (Solyc12g095750) and *SlAEC1* (Solyc02g037550) were zero; those of *SlAEC3* (Solyc02g091240), *SlAEC5* (Solyc03g032075), and *SlAEC6* (Solyc03g032080) did not show significant changes; and those of *SlAEC4* (Solyc03g031990) and *SlAEC7* (Solyc04g082830) showed slight changes between the WT and the mutants (**Figure 4A**). In addition, only the FPKM value of *SlPIN5* was downregulated in the two *slerf4-9* mutants among the 10 *SlPIN* genes (**Figure 4B**). The qRT-PCR results showed the same patterns (**Figure 4C**).

To investigate auxin transport in tomato roots, the DR5 strain was hybridized with the WT and the *slerf4-9* mutant. The results of the GUS staining assay suggested that the mutation of SlERF4-9 led to the reduced accumulation of auxin in LR primordia (**Figure 4D**). However, there were no GCC-box or DRE elements in the 5,000-bp promoters of *SlAEC2* and *SlPIN5*. Thus, SlERF4-9 may affect the efflux of auxin from intracellular to extracellular by indirectly regulating *SlAEC2* and *SlPIN5* expression and thereby improving LR development in tomatoes.

### SlERF4-9 regulates *SlCDF1* and *SlCDF3* expression by directly binding to their promoters

**Figure 5:**
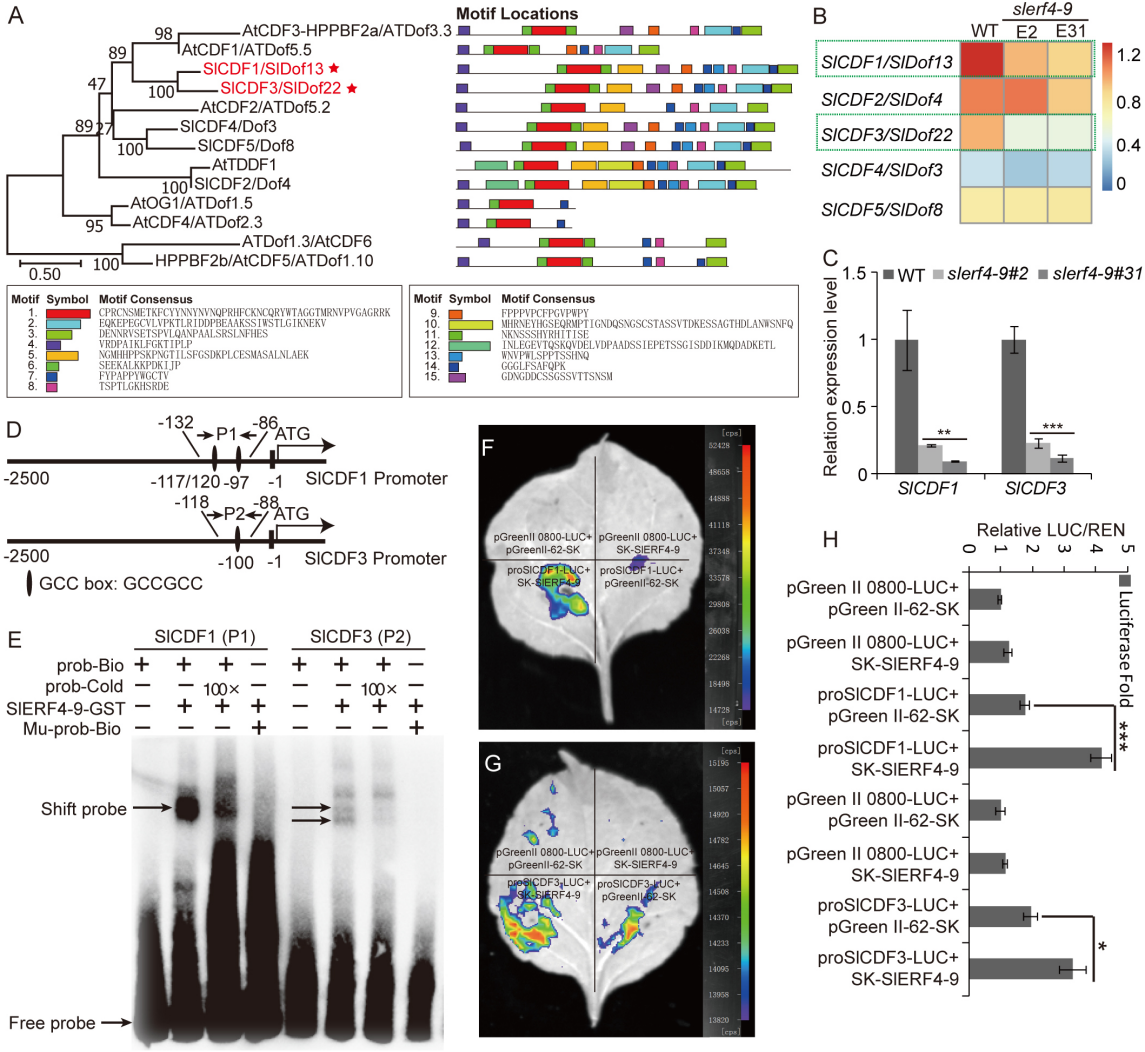
Regulatory relationship of SlERF4-9 and SlCDF1/3. **(A)** Phylogenetic and motif analyses of SlCDF1 and SlCDF3 proteins, **(B)** Heatmap analysis using the FPKM values of *SlCDF1* and *SlCDF3*. **(C)** qRT-PCR analysis of *SlCDF1* and *SlCDF3*. **(D)** GCC-box site analysis of *SlCDF1* and *SlCDF3* promoters. **(E)** The binding assays of SlERF4-9-GST protein with the P1 site of *SlCDF1* promoter and the P2 site of *SlCDF3* promoter. **(F, G)** Dual-luciferase transient expression assays of SlERF4-9 protein bind with the promoter of *SlCDF1* and *SlCDF3*. **(H)** Luciferase fold analysis of LUC/REN using the leaves injected in the previous step. Asterisks denote significance relative to WT (Student’s t-test; *P < 0.05, **p < 0.01 and ***p < 0.001).

In the downregulated DEG analysis, two DOF zinc finger proteins, *SlCDF1* (Solyc03g115940) and *SlCDF3* (Solyc06g069760), were identified in the *slerf4-9* mutants. The phylogenetic analysis indicated that the motif distributions of SlCDF1 and SlCDF3 proteins were similar to those of AtCDF1 and AtCDF3; moreover, SlCDF4 and SlCDF5 were similar to AtCDF2, and SlCDF2 was similar to AtTDDF1 (**Figure 5A**). The results suggest that CDFs with a similar motif distribution may have similar functions. In the transcriptome results, the FPKM values of *SlCDF1* and *SlCDF3* in two *slerf4-9* mutants were lower than those in the WT, and those of *SlCDF2*, *SlCDF4*, and *SlCDF5* were not significantly different between the WT and the mutants (**Figure 5B**). Moreover, the qRT-PCR results indicated that the *SlERF4-9* mutation reduced the expression of *SlCDF1* and *SlCDF3* in the young tomato roots (**Figure 5C**). In addition, the promoters of SlCDF1 and SlCDF3 had three and one GCC-boxes, respectively (**Figure 5D**). Therefore, we speculated that SlERF4-9 may directly regulate the transcription of *SlCDF1* and *SlCDF3*. In the EMSA, the SlERF4-9-GST fusion protein could bind with the GCC-boxes of the *SlCDF1* and *SlCDF3* promoters (**Figure 5E**), especially the *SlCDF1* promoter. The dual-luciferase transient expression assay showed that SlERF4-9 could positively activate the transcription of *SlCDF1* and *SlCDF3* (**Figures 5F, G**). In addition, the luciferase fold analysis showed that the SlERF4-9 protein enhanced LUC expression via the promoters of *SlCDF1* and *SlCDF3* (**Figure 5H**). Therefore, the results suggest that SlERF4-9 can positively regulate *SlCDF1* and *SlCDF3* expression by directly binding the GCC-boxes of their promoters.

### SlCDF1 and SlCDF3 positively regulate *SlAEC2* and *SlPIN5* expression

There are eight SlAEC and 10 SlPIN proteins in tomatoes, and there are eight AtPIN-like and seven AtPIN proteins in *Arabidopsis*. These proteins were subjected to a phylogenetic analysis. The results indicated that all PIN proteins were clustered in the I subgroup, while all SlAEC and AtPIN-like proteins were in the II subgroup (**Supplementary Figure S2A**). A protein sequence alignment found that all SlAEC and SlPIN proteins except SlAEC5 had both A and B domains, but SlPIN proteins had a long non-homologous sequence within the interval connecting the A and B domains (**Supplementary Figure S2B**). Thus, their A and B domains may be key for auxin binding and transport, while the non-homologous sequence between the A and B domains may be responsible for other functions.

**Figure 6:**
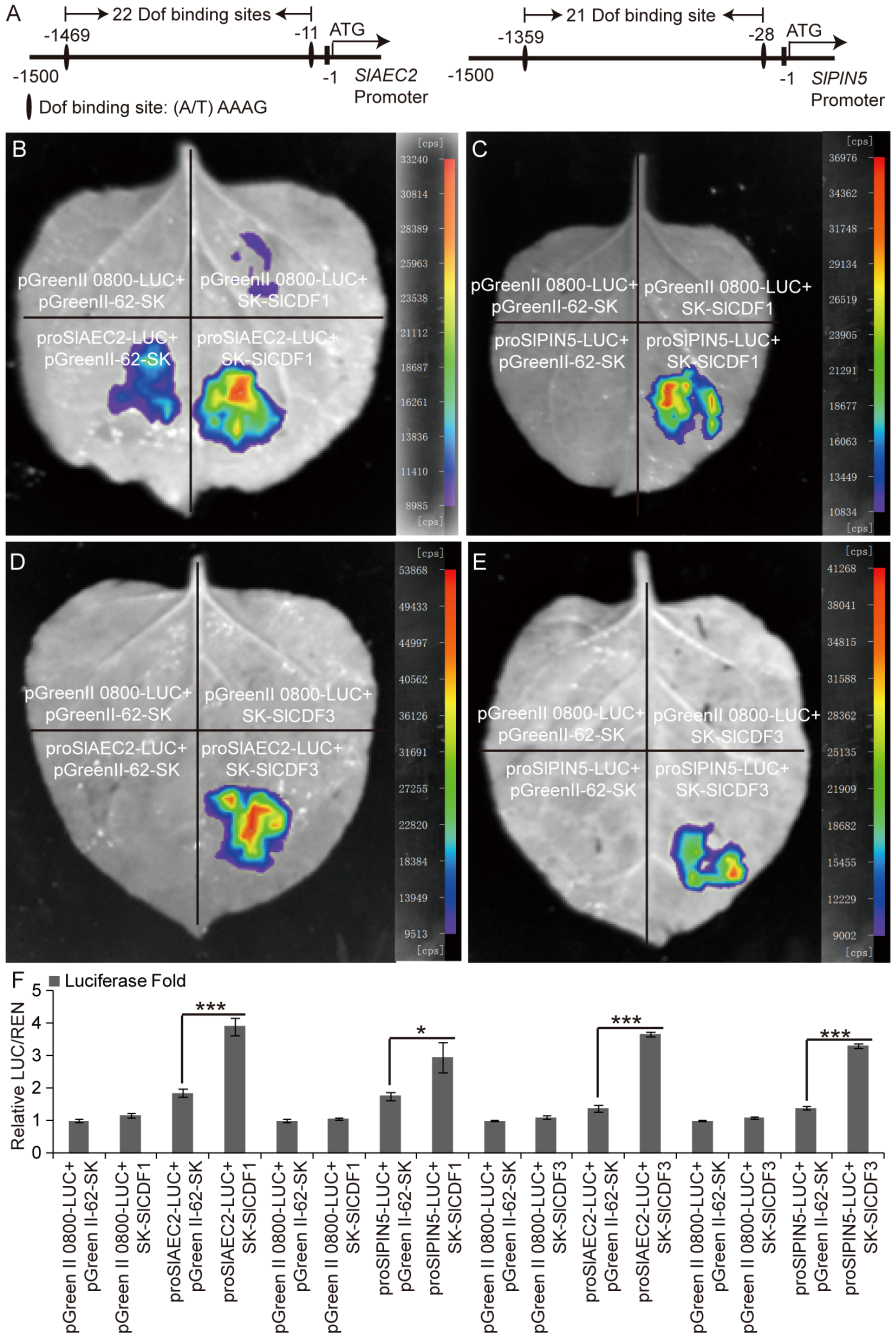
Regulatory relationship of SlCDF1/3 with SlAEC2 and SlPIN5. **(A)** DOF binding site analysis of *SlAEC2* and *SlPIN5* promoters. **(B–E)** The dual-luciferase transient expression assay of SlCDF1/3 protein binding with the promoter of *SlAEC2* and *SlPIN5*. **(F)** Luciferase fold analysis of LUC/REN using the leaves injected in the previous step. Three biological replicates were conducted in the assays. Asterisks denote significance relative to WT or control (Student’s t-test; *p < 0.05 and ***p < 0.001). DOF, DNA binding with One Finger.

There were 22 and 21 DOF transcription factor binding sites “(A/T)AAAG” on the *SlAEC2* and *SlPIN5* promoters, respectively (**Figure 6A**). We hypothesized that the transcription of *SlAEC2* and *SlPIN5* may be regulated by the SlCDF1 and SlCDF3 proteins. Therefore, a dual-luciferase transient expression assay was used to confirm this hypothesis. The results suggested that the transient expression of *SlCDF1* and *SlCDF3* activated LUC transcription through the *SlAEC2* and *SlPIN5* promoters (**Figures 6B–E**). A luciferase fold analysis yielded the same results (**Figure 6F**). Thus, SlCDF1 and SlCDF3 positively regulate *SlAEC2* and *SlPIN5* expression by binding to their promoters.

## Discussion

The roots are responsible for absorbing and transporting water and nutrients from the soil (Lal, 1979). Plants with more developed roots will have a greater ability to absorb and utilize water and nutrients. Phytohormone signal networks play important regulatory roles during root growth and development.

The biosynthesis, transport, and signal transduction of auxin involve *SlYUCCA*, *SlTAA*, *SlIAA*, *SlARF*, *SlAEC*, and *SlPIN* (Blilou et al., 2005; Cheng et al., 2006; Liscum and Reed, 2002; Stepanova et al., 2008). In this study, we found that the *SlERF4-9* mutation reduced tomato seed germination and the number of LRs (**Figures 1C,D**, 2C,F,G,I). The transcriptome analysis of young tomato roots showed that the transcript levels of genes related to auxin biosynthesis and signal transduction such as *SlYUCCA*, *SlTAA*, *SlIAA*, and *SlARF* were unchanged (**Supplementary Figure S1**), while those of *SlAEC2* and *SlPIN5* decreased in the *slerf4-9* mutant (**Figures 4A–C**). In *Arabidopsis*, lateral root initiation and root and hypocotyl growth were repressed in *atpin5* mutants, and low concentrations of IAA did not improve root growth (Mravec et al., 2009). Auxin accumulation was altered in the DR5 × WT and DR5 × *slerf4-9* hybrid seedling roots, especially in the LR primordia (**Figure 4D**). This indicated that *SlERF4-9* is not involved in regulating auxin biosynthesis or signal transduction but may regulate the transcription of genes related to auxin transport and thus affect LR development. Interestingly, the AP2/ERF binding motifs GCC-box and DRE elements have not been found on the *SlAEC2* and *SlPIN5* promoters, but 22 and 21 DOF binding sites existed on the *SlAEC2* and *SlPIN5* promoters (**Figure 6A**). Therefore, we speculated that there may be some DOF factors between SlERF4-9 and these binding sites, regulated by SlERF4-9 and positively regulating *SlAEC2* and *SlPIN5* transcription to affect auxin transport in tomato roots.

**Figure 7:**
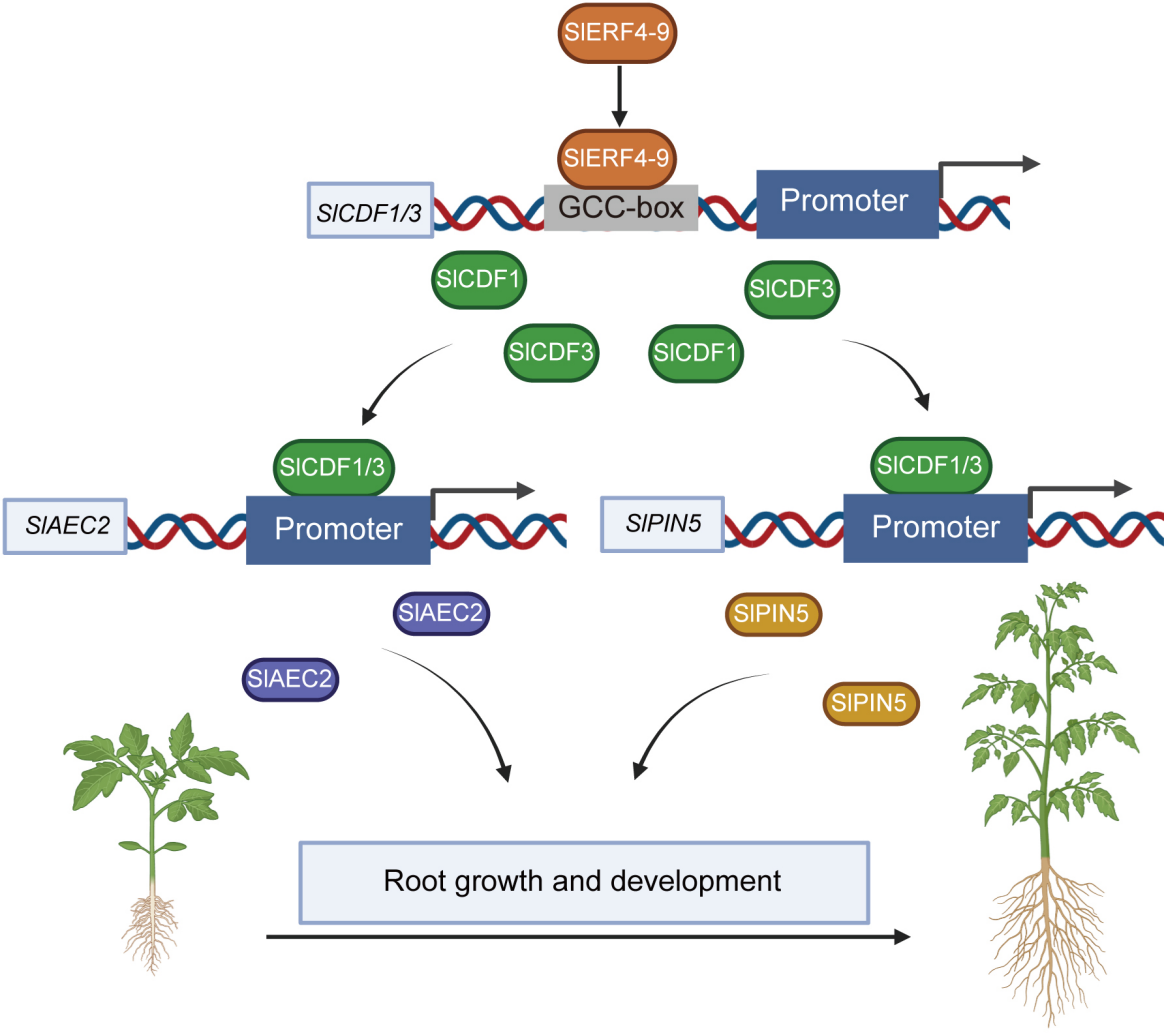
The regulatory network of SlERF4-9 involved in the growth and development of tomato roots.

The Cycling DOF Factors, a subgroup of the DOF family, regulate many plant life activities, including photoperiodic flowering, hypocotyl elongation, nitrogen assimilation, and abiotic stress responses (Corrales et al., 2017; Gao et al., 2022; Krahmer et al., 2019; Renau-Morata et al., 2020). In the DEG analysis of the transcriptome, the FPKM values of the two DOF factors *SlCDF1* and *SlCDF3* were reduced in *slerf4-9* mutant roots (**Figure 5B**). The qRT-PCR analysis yielded the same result (**Figure 5C**). Moreover, their respective promoters showed three and one GCC-box elements (**Figure 5D**). This suggested that the expression of *SlCDF1* and *SlCDF3* may be regulated by SlERF4-9. The results of EMSA and LUC assays showed that SlERF4-9 could interact with *SlCDF1* and *SlCDF3* promoters to positively regulate their transcription (**Figures 5E–H**). Studies have demonstrated that *SlCDF3* regulates flower timing, and its overexpression increases biomass production and yield (Renau-Morata et al., 2017; Xu et al., 2021). *AtCDF1* overexpression not only promotes the accumulation of carbon and nitrogen metabolites in potato tubers but also increases their yield (Carrillo et al., 2023). The phylogenetic and MEME analysis results suggested that SlCDF1, SlCDF3, AtCDF1, and AtCDF3 may have similar functions (**Figures 5A, B**). Therefore, reduced expression of *SlCDF1* and *SlCDF3* will inevitably affect the carbon and nitrogen metabolism in the roots, thereby affecting their growth and development. In addition, the results of the LUC assays suggested that both SlCDF1 and SlCDF3 could improve the transcription of *SlAEC2* and *SlPIN5* (**Figures 6B–F**). Therefore, SlCDF1 and SlCDF3 are involved in auxin transport by regulating *SlAEC2* and *SlPIN5* expression (**Figure 7**).

In conclusion, SlERF4-9 positively regulates *SlCDF1/3* transcription by binding to the GCC-boxes of their promoters; SlCDF1 and SlCDF3 improve the transcription of *SlAEC2* and *SlPIN5*; and SlAEC2 and SlPIN5 coordinate auxin transport and thereby affect the growth and development of tomato roots (**Figure 7**). The SlERF4-9-SlCDF1/3-SlAEC2/SlPIN5 model will provide important theoretical support for cultivating new tomato varieties with highly developed root systems.

## Data Availability

Raw data of RNA-seq was uploaded in the National Center for Biotechnology Information (NCBI) BioProject database with accession number PRJNA1091043.
