# Supplementary figures and images for: The SlERF4-9-SlCDF1/3-SlAEC2/SlPIN5 module regulates tomato root morphogenesis
Source: Front Plant Sci. 2025 Mar 28;16:1546092. doi: 10.3389/fpls.2025.1546092 (PMC11985770; doi:10.3389/fpls.2025.1546092)

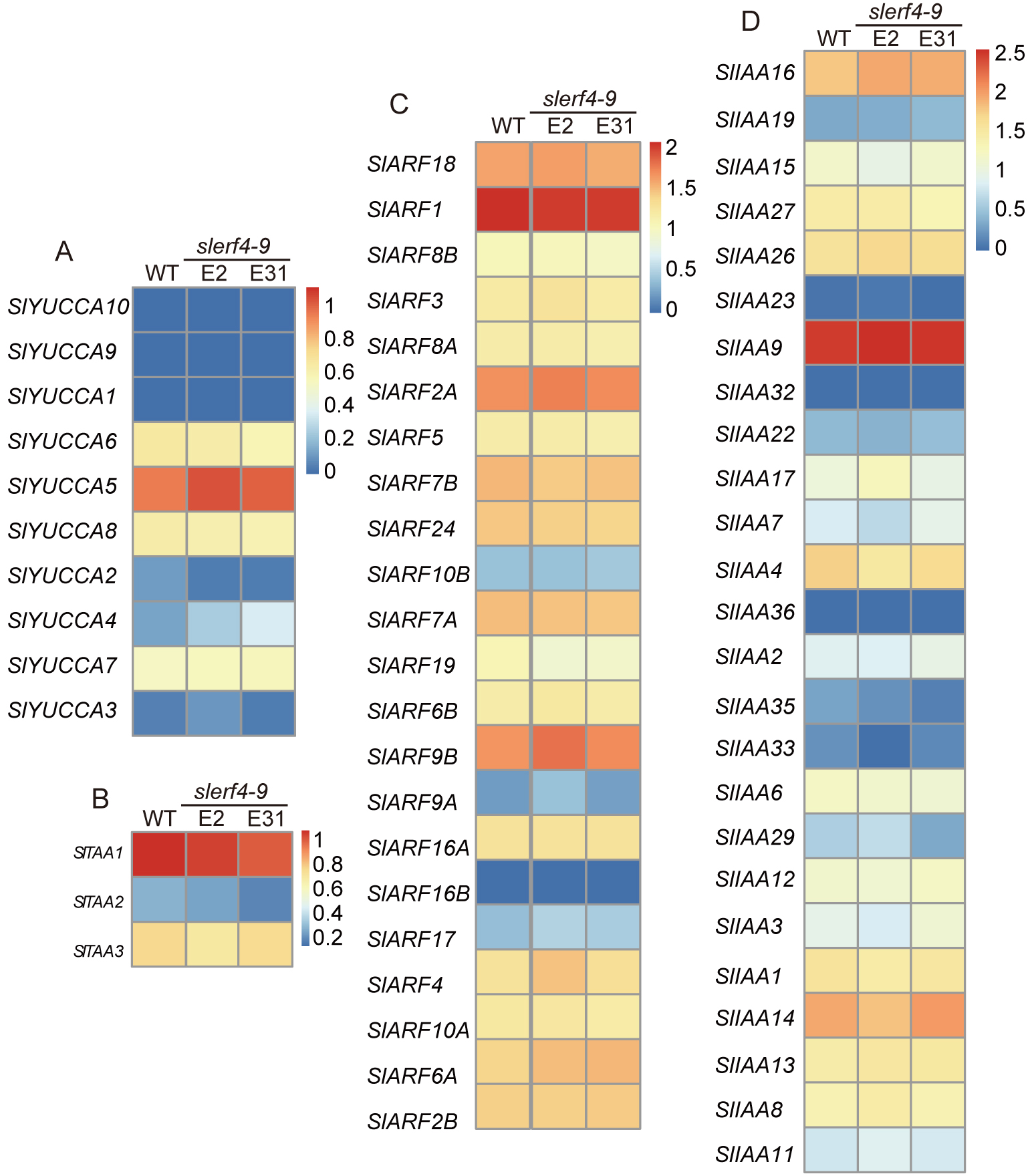

Supplement: Supplementary file 1 [file Image1.jpeg]

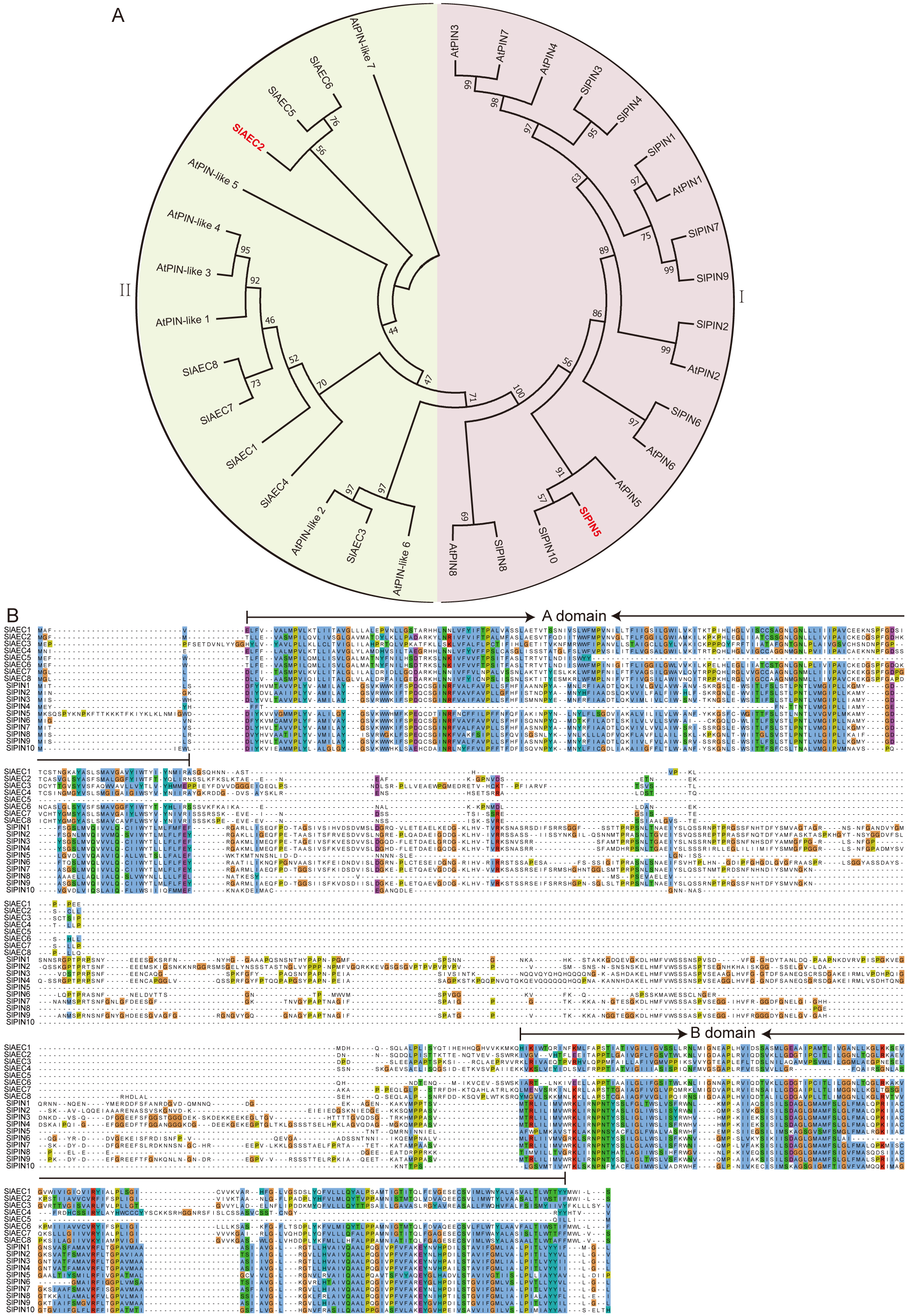

Supplement: Supplementary file 2 [file Image2.jpeg]
